# The personality factor in premium IOLs selection: quantifying Myers-Briggs personality types influence among cataract surgeons

**DOI:** 10.3389/fmed.2025.1710120

**Published:** 2025-12-03

**Authors:** Yinuo Wen, Yan Liu, Linghao Song, Xinyue Wang, Ruohong Li, Yue Yu, Shenjie Peng, Zexu Chen, Tianhui Chen, Yongxiang Jiang

**Affiliations:** 1 Eye Institute and Department of Ophthalmology, Eye & ENT Hospital, Fudan University, Shanghai, China; 2 Key laboratory of Myopia and Related Eye Diseases, NHC, Shanghai, China; 3 Key laboratory of Myopia and Related Eye Diseases, Chinese Academy of Medical Sciences, Shanghai, China; 4 Shanghai Key Laboratory of Visual Impairment and Restoration, Shanghai, China; 5 Shanghai Medical College (SHMC), Fudan University, Shanghai, China

**Keywords:** Myers-Briggs Type Indicator, personality types, cataract surgeons, intraocular lens selection, ocular parameters, demographics

## Abstract

**Background:**

Phacoemulsification with intraocular lens (IOL) implantation is the standard treatment for cataracts. The surgeon’s personality may influence IOL selection. Understanding whether Myers-Briggs Type Indicator (MBTI) personality profiles affect IOL selection accuracy and preferences can enhance patient satisfaction. Therefore, this retrospective single-center, cross-sectional study was conducted to evaluate the accuracy of IOL selection among cataract surgeons with different MBTI personality profiles and assess whether personality traits influence IOL preferences.

**Methods:**

A total of 50 cataract surgeons with different personality profiles were involved to select IOL for 43 patients based on collected preoperative data. Surgeons’ MBTI profiles were assessed, and their IOL selections were compared against clinical recommendations. Accuracy was analyzed across MBTI dichotomies (e.g., Extraversion/Introversion) and subtypes. Bland-Altman and consistency analyses evaluated preferences and agreement. Ocular biometric and comorbidity data were included.

**Results:**

No significant differences were found in IOL selection accuracy across the four MBTI dichotomous categories (*P* = 0.071, *P* = 0.178, *P* = 0.803, and *P* = 0.137, respectively). However, accuracy varied significantly across MBTI personality subtypes (*P* = 0.007). Consistency analysis revealed poor agreement across the four MBTI dimensions (Kappa = 0.012, Kappa = −0.013, Kappa = 0.001, and Kappa = 0.005, respectively). In contrast, Bland-Altman analysis indicated that Extraverted and Sensing surgeons showed a greater preference for multifocal and toric IOLs (all *P* < 0.05). Several ocular biometric parameters, ocular comorbidities, and systematic complications also significantly influenced the final choices (all *P* < 0.001).

**Conclusion:**

While MBTI personality profiles influenced IOL selection preferences, they did not significantly impact the precision of IOL decision-making. Clinical practice guidelines for IOL selection should be validated further, and MBTI could serve as a predictor for understanding surgeons’ IOL preferences. These findings suggest that IOL type preferences may vary among surgeons, which can help guide patients in choosing surgeons aligned with their specific needs.

## Introduction

Cataract remains the leading cause of severe visual impairment and may ultimately result in complete vision loss. Globally, more than 50 million individuals are affected by cataracts, and the prevalence continues to rise each year, posing a substantial burden on quality of life and reducing overall social productivity (1, 2). Advances in intraocular lens (IOL) have evolved beyond conventional monofocal IOLs, extending to bifocal, trifocal, toric, and extended depth of focus (EDOF) designs, offering surgeons a wide range of options to meet diverse patients’ needs (3). Consequently, when faced with multiple IOL choices, a surgeon’s personality traits may play a critical role in influencing both the accuracy and preference in selecting the most appropriate IOL for each patient (4).

The Myers-Briggs Type Indicator (MBTI) provides a scientifically informed and professionally nuanced framework for categorizing the personality profiles of medical practitioners. Developed in 1944, the MBTI has become one of the most widely used psychological assessment tools worldwide (5). It classifies individuals into 16 distinct personality types based on preferences across four dichotomous dimensions: Extraversion (E) versus (vs.) Introversion (I), Sensing (S) vs. Intuition (N), Thinking (T) vs. Feeling (F), and Judging (J) vs. Perceiving (P). By combining these eight preferences, the MBTI assigns each individual a unique personality type, providing valuable insight into their characteristic patterns of behavioral tendencies and problem-solving strategies (6).

Research on MBTI personality types within the medical field has attracted growing interest in recent years. Multiple international studies have explored the associations between MBTI profiles and various professional characteristics, like medical specialty, surgical training programs, clinical leadership, and workplace settings, among physicians and medical students worldwide (7–9). Systematic analyses have consistently shown that physicians demonstrate distinctive personality type distributions compared with the general population (7, 10, 11). Furthermore, several studies have identified correlations between MBTI types and the selection of specific medical specialties (12–14). However, it remains unclear whether certain personality types are more prevalent among ophthalmologists compared to other medical professionals. To date, no study has examined the relationship between ophthalmologists’ MBTI personality profiles and their IOL selection preferences, nor whether MBTI types could potentially predict surgeons’ patterns of IOL selection (15, 16).

In this study, we aim to investigate the relationship between cataract surgeons’ personality profiles and their IOL selection behaviors by comparing the accuracy and consistency of IOL choices among ophthalmologists with different MBTI types. Our findings seek to highlight the potential value of the MBTI as a complementary tool for both patients and surgeons, providing insights into how an ophthalmologist’s personality trait may influence the precision and preference in IOL selection.

## Materials and methods

### Patient eligibility and ethics statement

This retrospective, comparative, single-center, cross-sectional study was conducted in accordance with the Strengthening the Reporting of Observational Studies in Epidemiology (STROBE) guidelines. Written informed consent was obtained from all participants and from the legal guardians of those who under 18 years of age. The study was approved by the Human Research Ethics Committee of the Eye & ENT Hospital of Fudan University (ChiCTR2000039132) and adhered to the principles outlined in the Declaration of Helsinki.

### Participants and collection

This study employed a simulated IOL selection model in which cataract surgeons made IOL choices based on anonymized preoperative data from real patients. The simulation was designed to evaluate management decision accuracy without influencing actual clinical outcomes.

The inclusion criteria comprised cataract patients who underwent phacoemulsification with IOL implantation at Eye & ENT Hospital of Fudan University between March and July 2024. Patients with a history of ocular trauma or incomplete clinical data were excluded from the study. A total of 146 eyes from 74 patients were initially enrolled, 43 patients who completed all preoperative examinations were included in the final analysis, and one eye per patient was randomly chosen. All participants met the predefined inclusion criteria and provided written informed consent. The phacoemulsification and IOLs implantation procedures were performed by Dr. Yongxiang Jiang at the Eye and ENT Hospital of Fudan University, Shanghai, China.

Preoperative date collection included demographic characteristics, ocular history, ocular comorbidities, and relevant systematic medical histories. Best-corrected visual acuity (BCVA) and key biometric parameters were recorded, including the flattest Keratometry and steepest Keratometry (K1 and K2), axial length (AL), anterior chamber depth (ACD), lens thickness (LT), and white to white (WTW) distance. Ocular biometry was obtained using a combination of diagnostic tools: slit lamp examination, IOLMaster 700 (Carl Zeiss Meditec AG, Jena, Germany), B-scan ultrasonography (Aviso, Quantel Me’dical, Clermont-Ferrand, France), rotating Scheimpflug camera (Pentacam, Oculus Optikgeräte GmbH, Wetzlar, Germany), fundus photography (CLARUS 500, Carl Zeiss Meditec AG, Jena, Germany), and optical coherence tomography (OCT) (Spectrialis, Heidelberg Engineering, Heidelberg, Germany). BCVA values were converted to the logarithm of the minimal angle of resolution (logMAR) notation for analysis.

A modified version of Dell’s validated “Cataract and Refractive Lens Exchange Questionnaire” (CRLEQ) was utilized and adapted to reflect the institutional context and characteristics of the local patient population. The revised questionnaire included seven core domains beyond basic demographic information, covering patient’s reading habits, television-watching habits, driving habits, desire for spectacle independence, requirements for visual quality, and surgical method preference. The complete questionnaire is provided in Additional Material 1.

### Survey and procedure

A survey was distributed to 50 ophthalmologists in August 2024, including a link to the MBTI personality assessment, along with 80 additional questions covering demographic characteristics and for IOL selection preferences in 43 patients. Participation was voluntary, and all responses were kept strictly confidential. No financial compensation was provided. The questionnaire was submitted online and required approximately 2 hours to complete. IOL preferences were categorized into seven distinct types: monofocal IOLs, bifocal IOLs, trifocal IOLs, monofocal toric IOLs, bifocal toric IOLs, trifocal toric IOLs, and EDOF IOLs. EDOF IOLs in this study included both non-toric and toric models. It was important to note that no universal consensus currently existed in clinical practice regarding IOL selection criteria, resulting in considerable variability among surgeons’ surgical strategies. Therefore, in this study, the criteria for optimal IOL selection were established through a combination of evidence-based clinical guidelines and systematic evaluation by a panel of experienced ophthalmic surgeons, and referencing key international recommendations (17–20).

### MBTI assessment tool

The personality profiles of the 50 cataract surgeons were evaluated using the MBTI Form M, a validated psychological instrument designed to assess personality based on four dichotomous dimensions. The assessment was administered under the supervision of a certified psychologist. This version demonstrates acceptable internal consistency (Cronbach’s α = 0.76–0.84) and test-retest reliability (*r* = 0.73). The MBTI comprises 93 items that classify individuals across four primary dimensions, resulting in 16 distinct personality types. Each surgeon was assigned a corresponding MBTI type for subsequent analysis.

### Statistical analysis

All statistical analyses were performed using SPSS Statistics (version. 20.0, IBM Corp., Armonk NY, United States), with figures generated in GraphPad Prism 8 (GraphPad Software, San Diego, CA, United States) and R software (R Foundation for Statistical Computing, Vienna, Austria). IOL selection accuracy was defined as the proportion of surgeon-selected IOL types that matched the consensus “optimal IOL”, as determined by the expert panel based on evidence-based guidelines. Agreement in IOL preferences among MBTI personality types was evaluated using Cohen’s Kappa (κ) coefficient and visualized with Bland-Altman plots to determine the limits of agreement [mean difference ± 1.96 standard deviation (SD)], generated in MedCalc software (version 19.8; MedCalc Software Ltd., Ostend, Belgium).

Continuous variables were tested for normality using the Shapiro-Wilk test and expressed as mean ± SD for normally distributed data or median (interquartile range, IQR) for skewed data. Independent-samples *t*-tests or one-way ANOVA were used for normally distributed comparisons, while Mann-Whitney *U* or Kruskal-Wallis tests were applied for non-normally distributed data, with *post hoc* corrections for multiple comparisons where appropriate. Categorical variables were summarized as frequencies and percentages. Pearson’s χ^2^-test or Fisher’s exact test was used to compare categorical variables and to examine relationships between ocular biometric parameters and accuracy rates as well as inter-group comparisons of accuracy rates among MBTI categories. All tests were two-tailed, and *P* < 0.05 was considered statistically significant.

## Results

### Cohort characteristics

**FIGURE 1:**
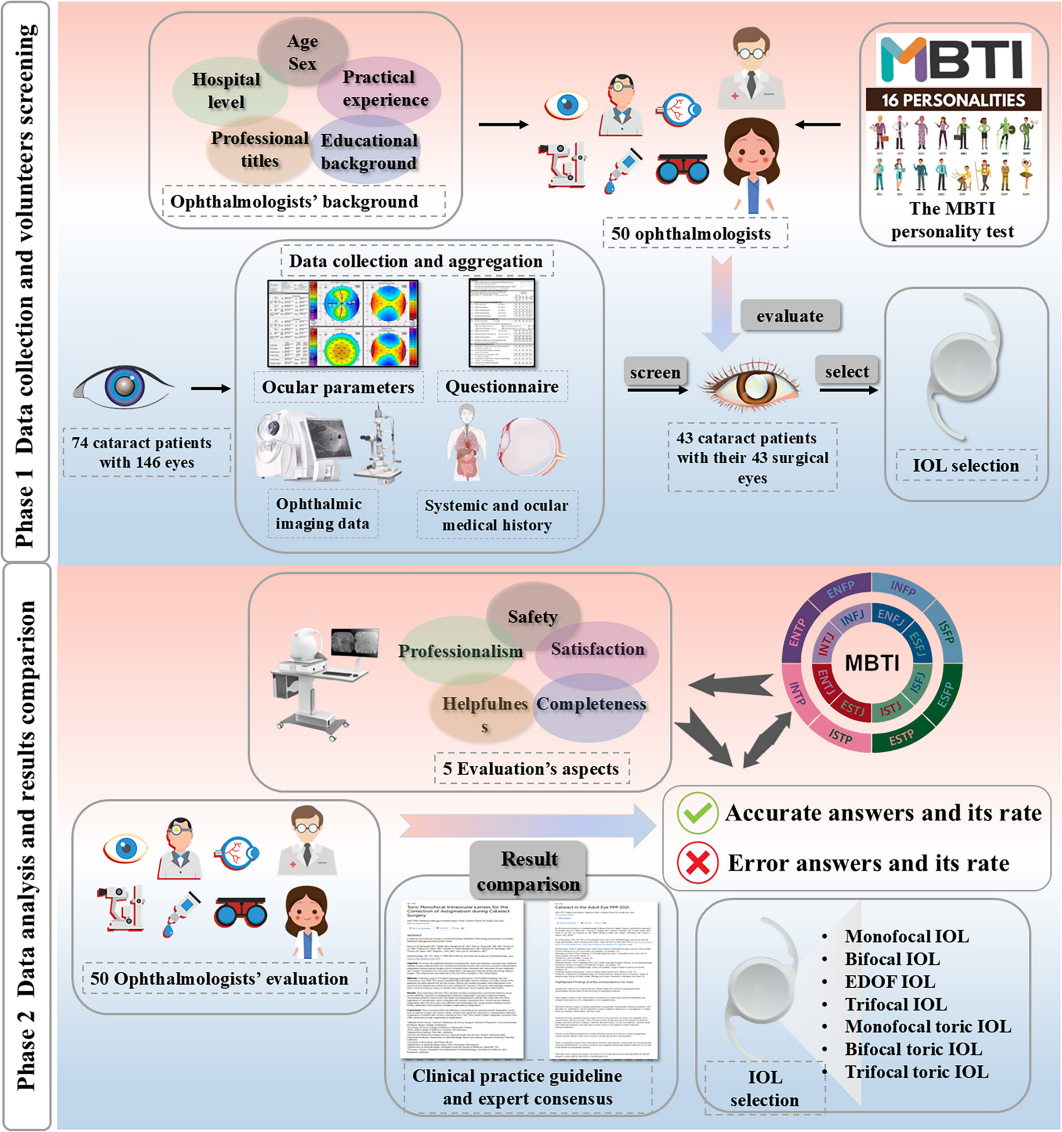
Study flowchart. This figure illustrates the two-phase methodology of our study: (1) assessment of MBTI personality profiles among cataract surgeons and (2) simulated IOL selection based on de-identified preoperative data from cataract patients obtained at a tertiary ophthalmic center. A total of 74 patients who underwent cataract evaluation were screened for eligibility, 43 patients with all follow-up assessments were selected for analysis, and one eye per patient was randomly chosen. The final dataset included 43 cataract patients with 43 surgical eyes for questionnaire validation. All subsequent analyses were based on the full cohort unless otherwise stated.

The workflow of this study is summarized in Figure 1. A total of 50 ophthalmologists completed the survey, among whom 68% were women (*N* = 34). Notably, 80% of respondents held senior professional titles, including Associate Chief Physician or Chief Physician (Additional Table 1). As illustrated in Additional Figure 1A, 48% of cataract surgeons were from eastern China (*N* = 24) (Additional Figure 1C). No statistically significant differences in demographic or professional characteristics, including gender, age, years of experience, surgical volume, familiarity with IOL technologies, professional rank, and educational background, were observed among the four MBTI dimensions (all *P* > 0.05; Additional Tables 2–5). These findings indicated that characteristics above were not confounding factors in the analysis of personality-related variations in IOL selection. Regarding MBTI profiles, 11 of the 16 personality types were represented in this cohort. The most common types were Introversion-Sensing-Thinking-Judging (ISTJ) (*N* = 9) and Introversion-Intuition-Thinking-Judging (INTJ) (*N* = 8). Less frequent types included Extraversion-Sensing-Feeling-Judging (ESFJ), Extraversion-Sensing-Feeling-Perceiving (ESFP), and Extraversion-Intuition-Feeling-Perceiving (ENFP), each accounting for 2% of participants (*N* = 1) (Additional Figure 1B). Owing to the small sample size, these three MBTI subtypes were excluded from subgroup analyses.

Additionally, 74 patients (146 eyes) were first enrolled. Baseline demographic characteristics were presented in Table 1. No statistically significant differences were observed between the left and right eyes for primary biometric ocular parameters, except for chord μ (*P* = 0.026) and chord α (*P* = 0.018).

**TABLE 1:** Demographic and ocular parameter information of the enrolled cataract patients.

Subjects	Mean ± SD (range) or median (IQR) or number (%)		
Number (N)	74		
Age (year)	61.24 ± 19.87		
Sex (male, %)	37 (46.20)		
Eye (right, %)	73 (50.00)		
**Ocular parameters**	**Right eyes (*N* = 41)**	**Left eyes (*N* = 33)**	***P-*value**
Axial length (mm)	24.29 (22.99, 26.26)	24.23 (23.03, 25.97)	0.799
K1 (D)	43.16 ± 2.02	43.15 ± 1.90	0.965
K1 axis (°)	95.00 ± 56.51	80 (27,118)	0.152
K2 (D)	44.34 ± 1.95	44.14 ± 1.93	0.538
K2 axis (°)	88.84 ± 52.56	92.75 ± 53.05	0.655
Astigmatism (D)	−0.92 (−1.48, −0.49)	−0.64 (−1.12, v0.41)	0.165
Anterior chamber depth (mm)	3.19 ± 0.70	3.11 ± 0.64	0.452
Lens thickness (mm)	4.35 (4.00, 4.83)	4.41 (4.07, 4.74)	0.638
White-to-white (mm)	11.80 (11.50, 12.10)	11.81 ± 0.53	0.918
Chord μ (mm)	0.26 (0.20, 0.38)	0.21 (0.13, 0.31)	**0.026^a^**
Chord α (mm)	0.40 (0.29, 0.50)	0.32 (0.26, 0.43)	**0.018^a^**
B/F ratio (%)	82.00 (81.00, 83.00)	81.7 (80.80, 83.59)	0.608
Pupil diameter (mm)	3.46 (3.08, 4.47)	3.34 (2.79, 3.82)	0.069
Total corneal HOA (μm)	0.21 (0.15, 0.29)	0.23 ± 0.17	0.369
Total corneal Z40 (μm)	0.30 ± 0.18	0.29 ± 0.15	0.801
UCVA	0.35 (0.10, 0.60)	0.35 (0.10, 0.60)	1.000
UCVA (logMAR)	0.46 (0.22, 1.00)	0.46 (0.22, 1.00)	1.000
BCVA	0.44 ± 0.33	0.44 ± 0.33	1.000
BCVA (logMAR)	0.39 (0.22, 0.82)	0.39 (0.22, 0.82)	1.000
Previous history of IOL implantation (yes, %) 6 (4.00)			
Ocular complications			
High myopia 9 (12.00)			
Macular degeneration 10 (13.00)			
Lens dislocation 5 (6.67)			
Uveitis 3 (4.00)			
Others 6 (8.00)			

B/F ratio, the mean radius of the posterior corneal surface to the mean radius of the anterior corneal surface; BCVA, best corrected visual acuity; D, diopter; K1, flattest keratometry; K2, steepest keratometry; LogMAR, logarithm of the minimum angle of resolution; Total corneal HOA, the total corneal higher-order aberrations calculated within the 4.0 mm zone around the corneal apex; Total corneal Z40, the total corneal spherical aberrations within the 6.0 mm zone around the corneal apex; UCVA, uncorrected visual acuity; Angle Kappa and angle Alpha are, respectively represented by chord μ and chord α, respectively, in the Scheimpflug cameras (Pentacam, Oculus Optikgeräte GmbH, Wetzlar, Germany). ^a^*P* < 0.05.

### The accuracy of IOL selection analysis

The accuracy of IOL selection among 50 ophthalmologists is presented in Additional Figure 2. The Extraversion group demonstrated the lowest accuracy rate (58.1%), whereas the Perceiving group showed a comparatively higher accuracy rate (66.7%) (Additional Figure 2A). To further examine the association between MBTI personality dimensions and IOL selection performance, accuracy ratios were calculated for each of the four dichotomous domains. No statistically significant difference was detected between E vs. I, S vs. I, T vs. F, or J vs. P groups with respect to accuracy in IOL selection (*P* = 0.071, *P* = 0.178, *P* = 0.803, and *P* = 0.137, respectively) (Table 2). However, a significant difference in IOL selection accuracy was observed across individual MBTI subtypes (χ2 = 2.783, *P* = 0.007). Specially, the Introversion-Intuition-Feeling-Perceiving (INFP) and ISTJ types exhibited the highest accuracy rates at 68.6% and 68.2%, respectively, whereas the Extraversion-Intuition-Thinking-Judging (ENTJ) subtype showed the lowest accuracy rate at 54.3% (Additional Figure 2B). *Post hoc* pairwise comparisons with multiple-testing correction among MBTI subtypes were presented in Additional Figure 2C.

**TABLE 2:** Accuracy rate, intergroup differences, and agreement in IOL selection across MBTI personality dimensions (*N* = 50 surgeons).

Characteristics	Accuracy rate (*N*, %)	Error rate (*N*, %)	χ2	*P*-value
Extraversion	200 (58.10)	144 (41.90)	−3.268	0.071
Introversion	1,143 (63.30)	663 (36.70)		
Sensing	498 (64.30)	276 (35.70)	1.815	0.178
Intuition	845 (61.40)	531 (38.60)		
Thinking	911 (62.30)	551 (37.70)	0.046	0.831
Feeling	432 (62.80)	256 (37.20)		
Judging	1,171 (61.90)	721 (38.10)	2.207	0.137
Perceiving	172 (66.70)	86 (33.30)		
**Characteristics**	**Correlation analysis**		**Consistency analysis**	
	**Pearson’s χ2-test**	***P*-value^a^**	**Kappa value**	***P*-value**
Extraversion	6.661	0.341	0.012	0.239
Introversion				
Sensing	3.069	0.803	−0.013	0.216
Intuition				
Thinking	5.632	0.468	0.001	0.93
Feeling				
Judging	4.793	0.559	0.005	0.261
Perceiving				

This table summarizes the IOL selection accuracy and intergroup statistical comparisons among eight major MBTI dimensions. Accuracy was defined as the proportion of selected IOLs matching the expert-determined optimal choice. Chi-square (χ2)-tests were used to compare categorical accuracy outcomes across MBTI groups. Cohen’s κ statistics assessed agreement levels within dichotomous MBTI dimensions. ^a^Chi-square comparison between eight dimensions’ groups of ophthalmologists.

### The correlation and the consistency of IOL selection analysis

**FIGURE 2:**
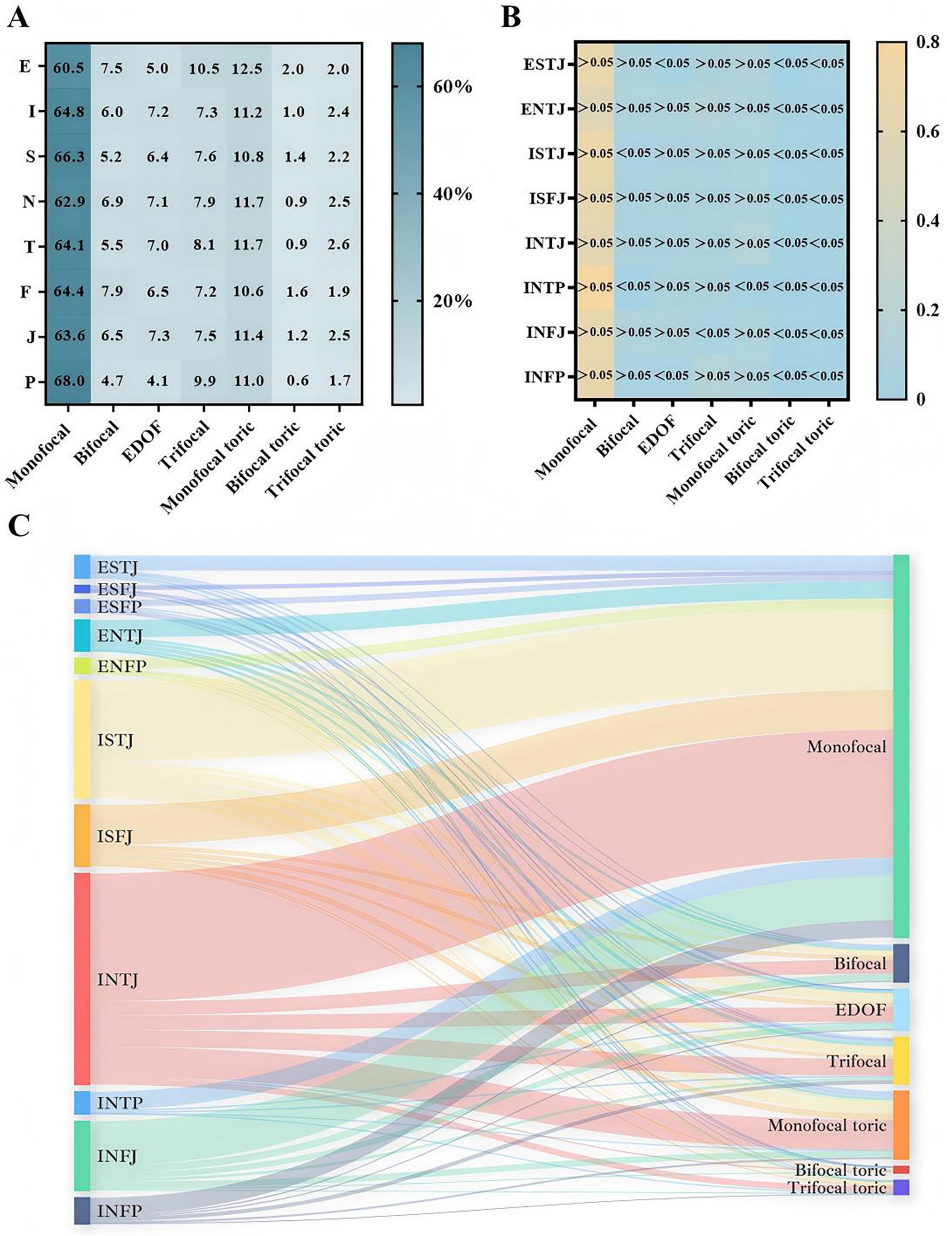
Distribution of IOL selection across MBTI types. **(A)** Heatmaps illustrating specific percentages of IOL types selected across eight MBTI dimensions. **(B)** Heatmaps showing proportional differences in IOL selection patterns among the eight major MBTI personality subgroups. **(C)** Sankey diagram demonstrating the relationships between eleven MBTI personality profiles and the seven IOL types. The thickness of each pathway represents the relative proportion of surgeons selecting a given IOL type. Monofocal IOLs were most frequently selected across personality subtypes, followed by monofocal toric IOLs.

The Sankey diagram illustrates the distribution of preferred IOL types across 50 cataract surgeons representing 11 distinct MBTI personality profiles (Figure 2C). Overall, monofocal IOLs were most frequently selected across all participants, followed by monofocal toric IOLs. No significant associations were detected between IOL selections and the four MBTI dimensions (E vs. I, S vs. N, T vs. F, and J vs. P) (*P* > 0.05; Figure 2A). To evaluate consistency within the MBTI dimensions, Cohen’s kappa coefficients were calculated, yielding values of 0.012, −0.013, 0.001, and 0.005, respectively (Table 2), all indicating minimal agreement. Bland-Altman analysis further demonstrated notable discrepancies between S- and T-type personality groups and their counterparts (Additional Figure 3). Moreover, the χ2 test (χ2 = 77.635, *P* < 0.001) and Kappa analysis (κ = 0.022, *P* = 0.032) confirmed significant variations in IOL preferences across MBTI subtypes. Subgroup analysis showed that INFP surgeons tended to prefer bifocal and trifocal options, ENTJ surgeons were more inclined toward EDOF and trifocal IOLs, and INTJ surgeons more frequently selected monofocal toric IOLs (Figure 2B).

### Relevant factors influencing IOL choices among different MBTI groups

**FIGURE 3:**
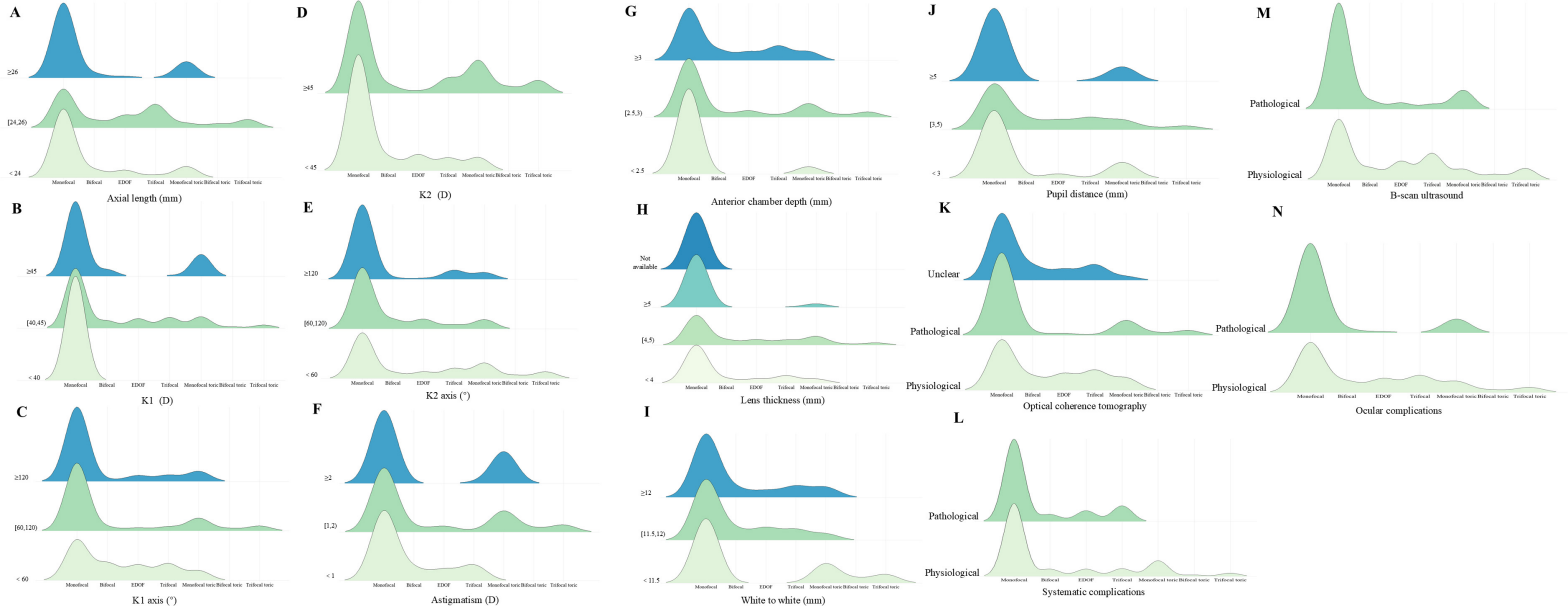
Accuracy distribution under different ocular conditions. Ridgeline density plots of IOL selection accuracy across clinical scenarios. Higher density indicates stronger agreement with the optimal IOL determined by the expert panel. **(A)** Distribution of IOL selection in patients under different axial lengths. **(B)** Distribution of IOL selection in patients under different K1 parameters. **(C)** Distribution of IOL selection in patients under different K1 axis. **(D)** Distribution of IOL selection in patients under different K2 parameters. **(E)** Distribution of IOL selection in patients under different K2 axis. **(F)** Distribution of IOL selection in patients under different corneal astigmatism. **(G)** Distribution of IOL selection in patients under different anterior chamber depths. **(H)** Distribution of IOL selection in patients under different lens thicknesses. **(I)** Distribution of IOL selection in patients under different white to white parameters. **(J)** Distribution of IOL selection in patients under different pupil distances. **(K)** Distribution of IOL selection in patients checked with optical coherence tomography. **(L)** Distribution of IOL selection in patients with or without systemic diseases. **(M)** Distribution of IOL selection in patients checked with B-scan ultrasound. **(N)** Distribution of IOL selection in patients with or without ocular complications.

Ridgeline plots were generated to visualize the distribution of accurate IOL selections under various clinical conditions. In patients with ocular comorbidities, monofocal and monofocal toric IOLs remained the most frequently preferred options. When both K1 and K2 values were <45 Diopter (D), K2 demonstrated less stringent influence on IOL selection than K1. Among eyes with corneal astigmatism ≥ 2 D, surgeons showed a stronger preference for monofocal and monofocal toric IOLs. Additionally, pupil diameter (PD) influenced decision-making, with monofocal, EDOF and monofocal toric IOLs being favored when PD < 3 mm (Figure 3). The interplay between ocular parameters and IOL preference varied across MBTI profiles. Univariate analysis indicated that IOL selection was not significantly associated with chord μ and α, B/F ratio, total corneal higher-order aberration (HOA), or total corneal spherical aberration (Z40) (all *P* > 0.05). However, after adjusting for confounders, a significant association emerged (*P* < 0.001), suggesting that personality traits might modulate the influence of ocular parameters on clinical judgments (Additional Table 6).

## Discussion

This study examined whether cataract surgeons’ MBTI personality profiles influenced the IOL selection by evaluating the distribution, accuracy, and consistency of their choices. Previous studies have reported that physicians displayed distinct MBTI personality patterns compared with the general population (7). Consistent with this, the majority of cataract surgeons in our cohort were classified as ISTJ or INTJ. Importantly, MBTI profiles were not associated with IOL selection accuracy across personality subtypes.

One possible explanation is that adherence to clinical practice guidelines (CPGs) may minimize subjective bias in IOL selection, regardless of personality traits. Our findings highlighted the critical role of CPGs in improving care quality and achieving optimal outcomes by equipping clinicians with evidence-based frameworks that balance clinical benefits with patients’ needs and values. In this way, CPGs might attenuate personality-driven variability in clinical decisions (21, 22).

Although no significant differences in selection accuracy were observed across MBTI categories, variability in consistency was noted across clinical scenarios. Each personality group demonstrated characteristic IOL selection tendencies. Zhao et al. suggested that the E-I dimension influenced how individuals engaged with their environment, with extraverts exhibiting a more outward-oriented and interactive style, whereas introverts tend to adopt a more reflective and internally focused approach (23). Similarly, our study indicated that introverted surgeons tended to prefer monofocal IOLs, whereas extraverted surgeons were more inclined to choose premium IOLs that supported greater spectacle independence. Furthermore, surgeons with sensing traits showed a preference for functional IOL models such as EDOF and toric IOLs. According to MBTI theory, sensing-dominant individuals focus on concrete details, while intuitive individuals prioritize abstract patterns and inferred insights (24). The expanding range of IOL technologies adds complexity to clinical practice, as each lens type presents distinct advantages and limitations (25). Monofocal IOLs remain a reliable, conservative option but fail to address near-vision demands (26), while presbyopia-correcting IOLs, may compromise contrast sensitivity and are less suitable for patients with ocular comorbidities (27). Surgeons with E- and S- profiles appeared more willing to accept visual trade-offs to achieve spectacle independence, whereas introverted and intuitive surgeons leaned toward more conservative options.

Additionally, our analyses revealed that ocular parameters moderated the relationship between MBTI personality traits and IOL choices. In cases involving extreme ocular measurements, surgeons gravitated toward simpler IOL designs, such as monofocal or monofocal toric IOLs. This observation aligned with previous evidence that biometric variables, such as AL, ACD, and keratometry, were strongly associated with refractive prediction error (28). Other ocular metrics, including WTW, LT, pupil diameter (PD), and astigmatism, have also been showed to influence IOL power calculation and selection (29). Therefore, appropriate IOL selection necessitated strict guideline adherence paired with individualized evaluation of ocular biometry, lifestyle requirements, and economic considerations. Multiple confounding factors, including surgeons’ and patients’ personality characteristics, might influence clinical judgments and visual outcomes (30). Future research should extend this work by incorporating personality assessments among larger surgeon cohorts and medical trainees to further elucidate how individual traits influence clinical judgment in ophthalmology.

This study has several certain limitations that should be considered when interpreting the findings. First, the sample size was limited, although the MBTI framework comprises 16 personality types, only eleven types were represented, and eight types with sufficient participants (≥ 3) were included in subgroup analyses. The absence of certain rare profiles may restrict the generalizability of the results, as unrepresented types may demonstrate distinct cognitive or behavioral tendencies. Larger, multi-center studies with more balanced MBTI distributions are needed to validate and extend these findings. Second, personality characteristics were assessed using a self-reported MBTI questionnaire, which might be subject to response bias and limited psychometric robustness. In addition, the cross-sectional study design only reflected surgeons’ personality traits at a single time point. Longitudinal studies are warranted to investigate the stability of personality traits over time and their potential influence on clinical practice (31, 32). Third, this study was conducted as a single institution and relied solely on the MBTI model. External validation using additional populations and complementary personality assessment tools, such as the Computer-Based Assessment for Sampling Personal Characteristics (CASPER) and the Situational Judgment Test (SJT), would help strengthen the generalizability of the findings and provide a more comprehensive understanding of personality-driven clinical behavior (33, 34).

Despite these limitations, this study is among the first to investigate the relationship between MBTI personality profiles and IOL selection in cataract surgery. In contrast to prior research that has primarily focused on how objective, patient-specific factors influence surgeons’ IOL preferences (4, 35), our work explores the extent to which ophthalmologists’ personality traits may shape both the accuracy of IOL selection and the choice of specific IOL types.

## Conclusion

This retrospective, single-center, cross-sectional study advances the theoretical understanding of IOL selection patterns among ophthalmologists with different MBTI personality profiles and provides practical insights into the role of intrinsic motivational factors. Our findings reinforce the value of CPGs, as a reliable and equitable framework for clinical practice, while also suggesting that individual personality traits may subtly shape surgeons’ choices within these established standards. Accordingly, patients may benefit from carefully selecting surgeons whose practice philosophy and communication style align with their own expectations. MBTI-based personality assessment may hold meaningful potential in medical practice, and future studies should further investigate the interactions between medical professionals’ personalities in other personality inventories and their choices in various ophthalmic settings. Such efforts may ultimately support more personalized IOL selection strategies, enhance postoperative visual rehabilitation, and better meet patients’ increasing expectations for tailored and high-quality surgical outcomes.

## Data Availability

The original contributions presented in this study are included in this article/Supplementary material, further inquiries can be directed to the corresponding authors.
