# Supplementary material for: The personality factor in premium IOLs selection: quantifying Myers-Briggs personality types influence among cataract surgeons
Source: Front Med (Lausanne). 2025 Dec 3;12:1710120. doi: 10.3389/fmed.2025.1710120 (PMC12708886; doi:10.3389/fmed.2025.1710120)
Supplement: Supplementary file 1 [file Data_Sheet_1.docx]

**
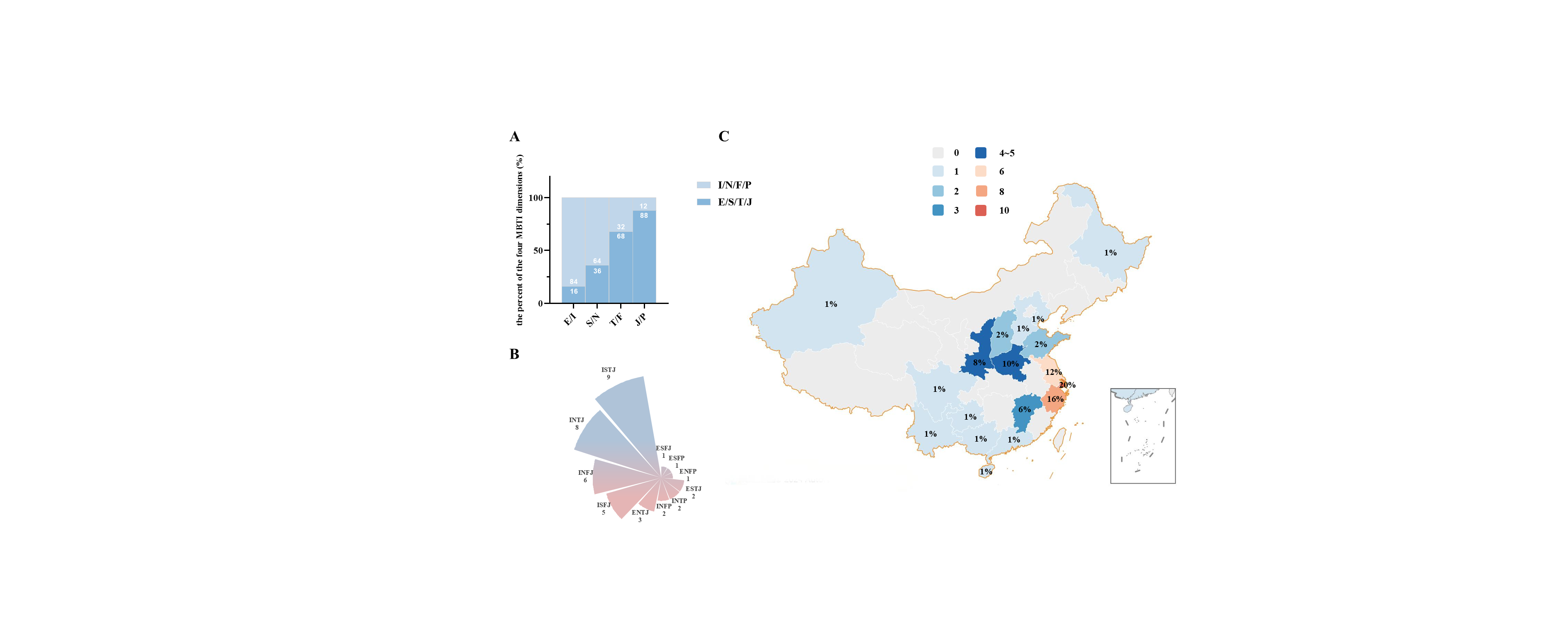
Additional Figure 1: Surgeon demographic and background profiles**.

1. The percentage of ophthalmologists in each of the eight dimensions of MBTI. **B.** The specific numbers of the corresponding eleven MBTI personality profiles for ophthalmologists. **C.** The geographical distribution of the ophthalmologists.


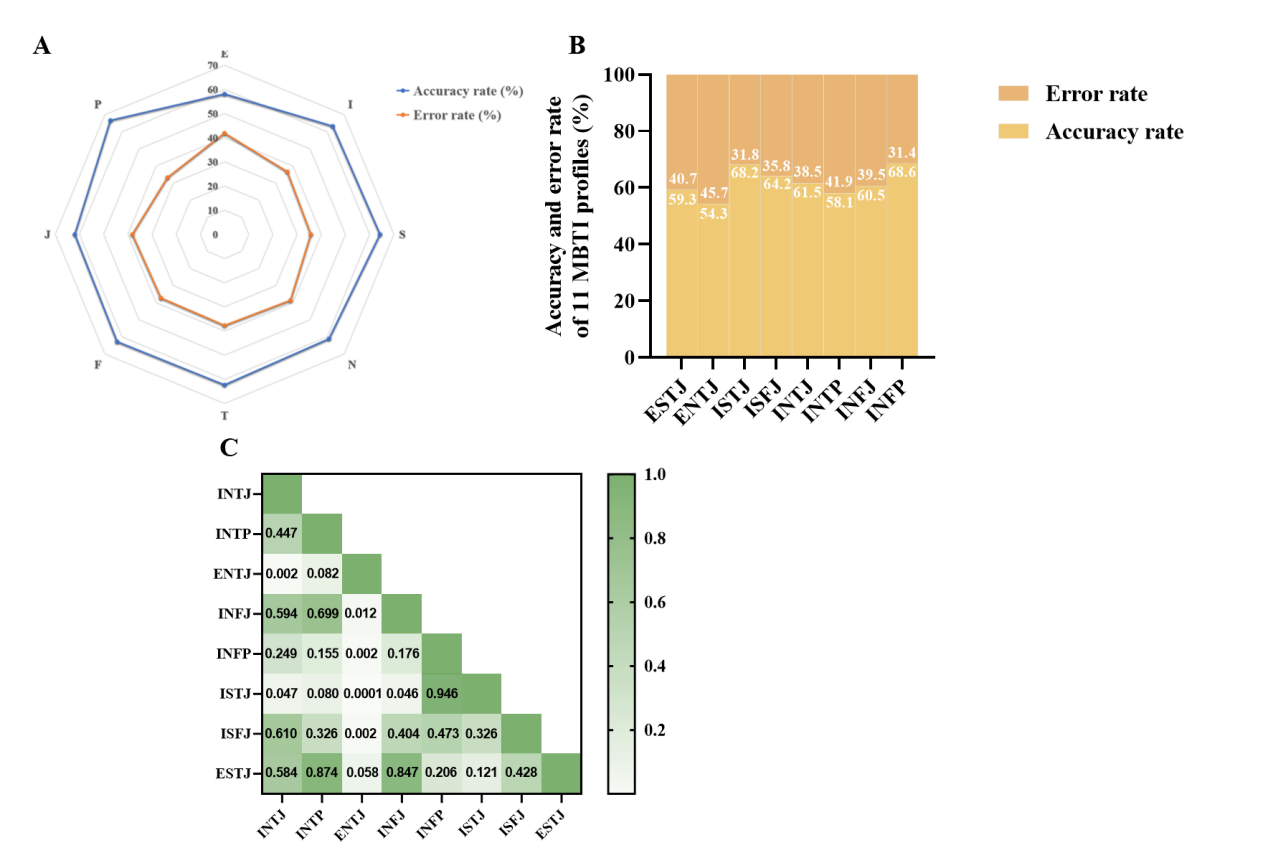


**Additional Figure 2: Comparison of accuracy across MBTI subgroups.**

Accuracy rates stratified by MBTI dichotomies and cognitive functions across multiple simulated clinical scenarios. **A.** Comparison of ophthalmologists’ accuracy in patients from eight dimensions across different IOL selection. **B.** Comparison of ophthalmologists’ accuracy from eight MBTI personality types across different IOL selection. **C.** Post-hoc corrections for multiple comparisons of ophthalmologists’ accuracy from eight MBTI personality types across different IOL selection.


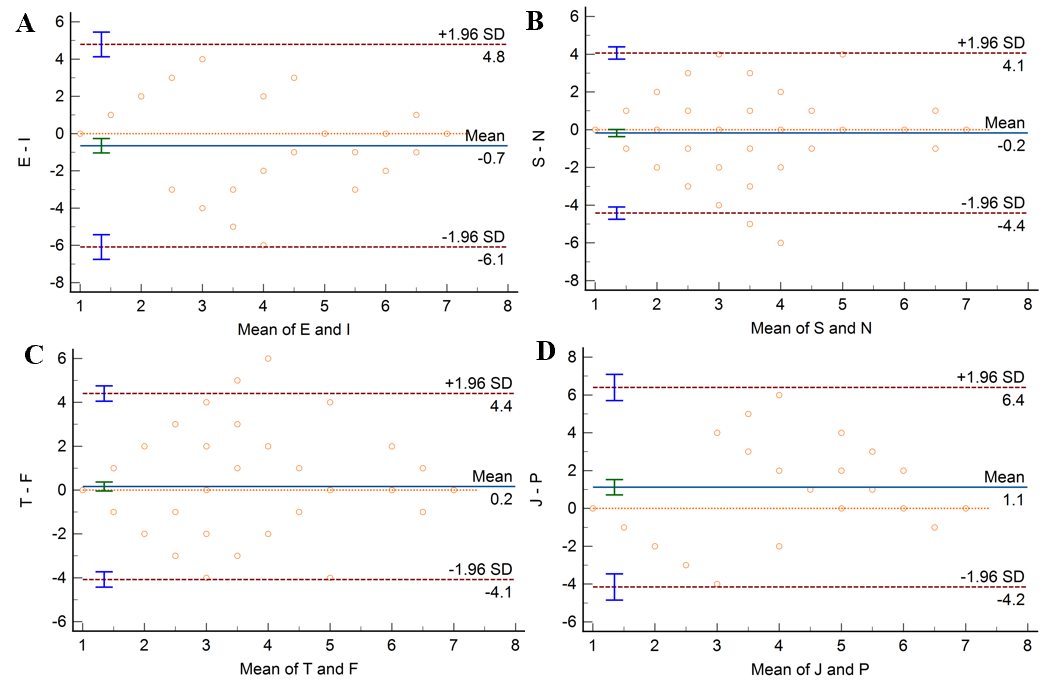


**Additional Figure 3: Bland-Altman consistency analysis among MBTI dimensions.**

Agreement of IOL selection accuracy between E vs. I, S vs. N, T vs. F, and J vs. P dimensions. **A.** Comparison of the overall consistency in accuracy between E and I in IOL selection across multiple tests. **B.** Comparison of the consistency in accuracy between S and I in IOL selection across multiple tests. **C.** Comparison of the consistency in accuracy between T and F in IOL selection across multiple tests. **D.** Comparison of the consistency in accuracy between J and P in IOL selection across multiple tests.
