# Supplementary material for: The personality factor in premium IOLs selection: quantifying Myers-Briggs personality types influence among cataract surgeons
Source: Front Med (Lausanne). 2025 Dec 3;12:1710120. doi: 10.3389/fmed.2025.1710120 (PMC12708886; doi:10.3389/fmed.2025.1710120)
Supplement: Supplementary file 2 [file Data_Sheet_2.docx]

**Additional Table 1: Basic information and related data of participating ophthalmologists.**

| **Characteristics** | **Mean ± SD (Range) or Median (IQR) or Number (%)** | |
| --- | --- | --- |
| Number (N) | 50 | |
| Sex (N, %) | Male | 16（32.00） |
|  | Female | 34（68.00） |
| Hospital level (N, %) | 2B | 3（6.00） |
|  | 2A | 5（10.00） |
|  | 3B | 6（12.00） |
|  | 3A | 36（72.00） |
| Age (years) | 43.22 ± 6.482 | |
| Experience (years) | 11.44 ± 7.885 | |
|  | ≤ 10 years in practice | 29（58.00） |
|  | ＞ 10 years in practice | 21（42.00） |
| Training and surgical volume (N, %) | 4707.80 ± 4984.474 | |
|  | ≤ 3000 cases | 24 （48.00） |
|  | ＞ 3000 cases | 26 （52.00） |
| Familiarity with current IOL technologies (N, %) | Novice | 12 （24.00） |
|  | Competent | 14 （28.00） |
|  | Proficient | 15 （30.00） |
|  | Expert | 9 （18.00） |
| Professional titles (N, %) | Attending physician | 10（20.00） |
|  | Associate chief physician | 22（44.00） |
|  | Chief physician | 18（36.00） |
| Educational background (N, %) | Bachelor's degree | 15（30.00） |
|  | Master's degree | 24（48.00） |
|  | Doctor’s degree | 11（22.00） |

**Additional Table 2: Frequency and percentage of basic information and related data of participating ophthalmologists in Extraversion/ Introversion profiles.**

| **Characteristics** | **Mean ± SD (Range) or Median (IQR) or Number (%)** | | |
| --- | --- | --- | --- |
|  | **Extraversion Introversion** | | ***P* value** |
| Number (N) | 8 | 42 | */* |
| Sex (N, %) | | | 0.382 |
| Male | 3 (33.30) | 13 (31.00) |  |
| Female | 5 (55.60) | 29 (69.00) |  |
| Hospital level (N, %) | | | 0.243 |
| 2B | 0 (0.00) | 3 (7.10) |  |
| 2A | 1 (12.50) | 4 (9.50) |  |
| 3B | 0 (0.00) | 6 (14.30) |  |
| 3A | 7 (87.50) | 29 (69.00) |  |
| Age (years) | 46.00 ± 6.85  (36, 56) | 42.69 ± 6.36  (30, 54) | 0.068 |
| Experience (years) | 15.00 ± 9.86  (1, 34) | 10.76 ± 7.40  (1, 28) | 0.085 |
| ≤ 10 years in practice | 3 (37.50) | 26 (61.90) |  |
| ＞ 10 years in practice | 5 (62.50) | 16 (38.10) |  |
| Training and surgical volume (N, %) | 6587.50 ± 6235.03  (1000, 10000) | 4349.76 ± 4715.38  (800, 7250) | 0.190 |
| ≤ 3000 cases | 2 (25.00) | 21 (50.00) |  |
| ＞ 3000 cases | 6 (75.00) | 21 (50.00) |  |
| Familiarity with current IOL technologies (N, %) | | | 0.103 |
| Novice | 1 (12.50) | 11 (26.19) |  |
| Competent | 2 (25.00) | 12 (28.57) |  |
| Proficient | 1 (12.50) | 14 (33.33) |  |
| Expert | 4 (50.00) | 5 (11.90) |  |
| Professional titles (N, %) | | | 0.187 |
| Attending physician | 1 (12.50) | 9 (21.43) |  |
| Associate chief physician | 3 (37.50) | 19 (45.23) |  |
| Chief physician | 4 (50.00) | 14 (33.33) |  |
| Educational background (N, %) | | | 0.180 |
| Bachelor's degree | 2 (25.00) | 13 (30.95) |  |
| Master's degree | 3 (37.50) | 21 (50.00) |  |
| Doctor’s degree | 3 (37.50) | 8 (19.05) |  |

**Additional Table 3: Frequency and percentage of basic information and related data of participating ophthalmologists in Sensing/ Intuition profiles.**

| **Characteristics** | **Mean ± SD (Range) or Median (IQR) or Number (%)** | | |
| --- | --- | --- | --- |
|  | **Sensing Intuition** | | ***P* value** |
| Number (N) | 18 | 32 | / |
| Sex (N, %) | | | 0.271 |
| Male | 4 (22.00) | 12 (37.50) |  |
| Female | 14 (77.80) | 20 (62.50) |  |
| Hospital level (N, %) | | | 0.219 |
| 2B | 1 (5.60) | 2 (6.30) |  |
| 2A | 1 (5.60) | 4 (12.50) |  |
| 3B | 1 (5.60) | 5 (15.60) |  |
| 3A | 15 (83.30) | 21 (65.60) |  |
| Age (years) | 43.61 ± 5.76  (32,56) | 43.00 ± 6.94  (30, 54) | 0.753 |
| Experience (years) | 12.33 ± 8.30  (1,34) | 10.94 ± 7.73  (1, 28) | 0.577 |
| ≤ 10 years in practice | 11 (61.10) | 18 (56.30) |  |
| ＞ 10 years in practice | 7 (38.90) | 14 (43.70) |  |
| Training and surgical volume (N, %) | 4552.78 ± 3732.47  (725, 8250) | 4795 ± 5622.24  (1000, 7750) | 0.149 |
| ≤ 3000 cases | 6 (33.33) | 18 (56.25) |  |
| ＞ 3000 cases | 12 (66.67) | 14 (43.75) |  |
| Familiarity with current IOL technologies (N, %) | | | 0.638 |
| Novice | 5 (27.78) | 7 (21.88) |  |
| Competent | 2 (11.11) | 12 (37.50) |  |
| Proficient | 8 (44.44) | 7 (21.88) |  |
| Expert | 3 (16.67) | 6 (18.75) |  |
| Professional titles (N, %) | | | 0.640 |
| Attending physician | 3 (16.67) | 7 (21.88) |  |
| Associate chief physician | 8 (44.44) | 14 (43.75) |  |
| Chief physician | 7 (38.89) | 11 (34.38) |  |
| Educational background (N, %) | | | 0.168 |
| Bachelor's degree | 4 (22.22) | 11 (34.38) |  |
| Master's degree | 8 (44.44) | 16 (50.00) |  |
| Doctor’s degree | 6 (33.33) | 5 (15.63) |  |

**Additional Table 4: Frequency and percentage of basic information and related data of participating ophthalmologists in Thinking/ Feeling profiles.**

| **Characteristics** | **Mean ± SD (Range) or Median (IQR) or Number (%)** | | |
| --- | --- | --- | --- |
|  | **Thinking Feeling** | | ***P* value** |
| Number (N) | 34 | 16 | / |
| Sex (N, %) | | | 0.173 |
| Male | 13 (38.20) | 3 (18.80) |  |
| Female | 21 (61.80) | 13 (81.30) |  |
| Hospital level (N, %) | | | 0.732 |
| 2B | 2 (5.90) | 1 (6.30) |  |
| 2A | 4 (11.80) | 1 (6.30) |  |
| 3B | 4 (11.80) | 2 (12.50) |  |
| 3A | 24 (70.60) | 12 (75.00) |  |
| Age (years) | 43.26 ± 6.64  (31,54) | 43.13 ± 6.34  (30, 56) | 0.944 |
| Experience (years) | 11.71 ± 7.82  (1,28) | 10.88 ± 8.25  (3,34) | 0.631 |
| ≤ 10 years in practice | 19 (55.90) | 10 (62.50) |  |
| ＞ 10 years in practice | 15 (44.10) | 6 (37.50) |  |
| Training and surgical volume (N, %) | 4966.18 ± 4976.32  (1000, 8000) | 4158.75 ± 5119.29  (575, 8000) | 0.983 |
| ≤ 3000 cases | 15 (44.12) | 9 (56.25) |  |
| ＞ 3000 cases | 19 (55.88) | 7 (43.75) |  |
| Familiarity with current IOL technologies (N, %) | | | 0.974 |
| Novice | 8 (23.53) | 4 (25.00) |  |
| Competent | 9 (26.47) | 5 (31.25) |  |
| Proficient | 12 (35.29) | 3 (18.75) |  |
| Expert | 5 (14.71) | 4 (25.00) |  |
| Professional titles (N, %) | | | 0.729 |
| Attending physician | 7 (20.59) | 3 (18.75) |  |
| Associate chief physician | 14 (41.18) | 8 (50.00) |  |
| Chief physician | 13 (38.24) | 5 (31.25) |  |
| Educational background (N, %) | | | 0.184 |
| Bachelor's degree | 11 (32.35) | 4 (25.00) |  |
| Master's degree | 18 (52.94) | 6 (37.50) |  |
| Doctor’s degree | 5 (14.71) | 6 (37.50) |  |

**Additional Table 5: Frequency and percentage of basic information and related data of participating ophthalmologists in Judging/ Perceiving profiles.**

| **Characteristics** | **Mean ± SD (Range) or Median (IQR) or Number (%)** | | |
| --- | --- | --- | --- |
|  | **Judging Perceiving** | | ***P* value** |
| Number (N) | 44 | 6 | / |
| Sex (N, %) | | | 0.941 |
| Male | 14 (31.80) | 2 (33.30) |  |
| Female | 30 (68.20) | 4 (66.70) |  |
| Hospital level (N, %) | | | 0.112 |
| 2B | 3 (6.80) | 0 (0.00) |  |
| 2A | 5 (11.40) | 0 (0.00) |  |
| 3B | 6 (13.60) | 0 (0.00) |  |
| 3A | 30 (68.20) | 4 (100.00) |  |
| Age (years) | 43.32 ± 6.63  (30,56) | 42.50 ± 5.75  (33, 49) | 0.775 |
| Experience (years) | 11.91 ± 7.96  (1,34) | 8.00 ± 7.00  (1, 18) | 0.214 |
| ≤ 10 years in practice | 25 (56.80) | 4 (66.70) |  |
| ＞ 10 years in practice | 19 (43.20) | 2 (33.30) |  |
| Training and surgical volume (N, %) | 4617.95 ± 4752.31 (1000, 8000) | 5366.67 ± 6975.86  (650, 11250) | 0.203 |
| ≤ 3000 cases | 21 (47.73) | 3 (50.00) |  |
| ＞ 3000 cases | 23 (52.27) | 3 (50.00) |  |
| Familiarity with current IOL technologies (N, %) | | | 0.902 |
| Novice | 11 (25.00) | 1 (16.67) |  |
| Competent | 11 (25.00) | 3 (50.00) |  |
| Proficient | 15 (34.09) | 0 (0.00) |  |
| Expert | 7 (15.91) | 2 (33.33) |  |
| Professional titles (N, %) | | | 0.987 |
| Attending physician | 9 (20.45) | 1 (16.67) |  |
| Associate chief physician | 19 (43.18) | 3 (50.00) |  |
| Chief physician | 16 (36.36) | 2 (33.33) |  |
| Educational background (N, %) | | | 0.146 |
| Bachelor's degree | 14 (31.82) | 1 (16.67) |  |
| Master's degree | 22 (50.00) | 2 (33.33) |  |
| Doctor’s degree | 8 (18.18) | 3 (50.00) |  |

**Additional Table 6:** **Analysis of the correlation between ocular parameter information and IOL selection in accurate answers among ophthalmologists.**
